# B Cells Are Indispensable for a Novel Mouse Model of Primary Sjögren’s Syndrome

**DOI:** 10.3389/fimmu.2017.01384

**Published:** 2017-10-24

**Authors:** Junfeng Zheng, Qiaoniang Huang, Renliang Huang, Fengyuan Deng, Xiaoyang Yue, Junping Yin, Wenjie Zhao, Yan Chen, Lifang Wen, Jun Zhou, Renda Huang, Gabriela Riemekasten, Zuguo Liu, Frank Petersen, Xinhua Yu

**Affiliations:** ^1^Xiamen-Borstel Joint Laboratory of Autoimmunity, Medical College of Xiamen University, Xiamen, China; ^2^Institute of Psychiatry and Neuroscience, Xinxiang Medical University, XinXiang, China; ^3^Priority Area Asthma & Allergy, Research Center Borstel, Airway Research Center North (ARCN), Members of the German Center for Lung Research (DZL), Borstel, Germany; ^4^Department of Rheumatology, University of Lübeck, Lübeck, Germany; ^5^Eye Institute of Xiamen University, The Medical College of Xiamen University, Xiamen, China

**Keywords:** primary Sjögren’s syndrome, mouse model, T cell epitope, SSA, autoantibodies, B cells

## Abstract

Primary Sjögren’s syndrome (pSS) is characterized by a panel of autoantibodies, while it is not clear whether B cells and autoantibodies play an essential role in pathogenesis of the disease. Here, we report a novel mouse model for pSS which is induced by immunization with the Ro60_316-335 peptide containing a predominant T cell epitope. After immunization, mice developed several symptoms mimicking pSS, including a decreased secretion of tears, lymphocytic infiltration into the lacrimal glands, autoantibodies, and increased levels of inflammatory cytokines. Disease susceptibility to this novel mouse model varies among strains, where C3H/HeJ (H2-k) and C3H/HeN (H2-k) are susceptible while DBA/1 (H2-q) and C57BL/6 (H2-b) are resistant. Depletion of B cells using anti-CD20 monoclonal antibodies prevented C3H/HeN mice from development of the pSS-like disease. In addition, HLA-DRB1*0803, a pSS risk allele, was predicted to bind to the hRo60_308-328 which contains a predominant T cell epitope of human Ro60. Therefore, this study provides a novel mouse model for pSS and reveals an indispensable role of B cells in this model. Moreover, it suggests that T cell epitope within Ro60 antigen is potentially pathogenic for pSS.

## Introduction

Primary Sjögren’s syndrome (pSS) is an autoimmune disorder mainly targeting salivary and lacrimal glands and leading to xerostomia (dry mouth) and xerophthalmia (dry eye) (1). The pSS is characterized by lymphocytic infiltrates into the exocrine glands as well as by a specific panel of circulating autoantibodies (2). In contrast to the unknown disease-related autoreactive T cells, many autoantibodies have been identified in patients with pSS, including anti-SSA/Ro and anti-SSB/La autoantibodies, rheumatoid factor, anti-nuclear antibody, anti-muscarinic type 3 acetylcholine receptors (M3R), and anti-α fodrin antibodies (3). Although B cell hyper-activation resulting hypergammaglobulinemia and production of autoantibodies is a predominant feature of pSS, it is not clear whether B cells and autoantibodies are indispensable for the development of the disease. Clinical evidence from B cell-targeting therapy show an inconclusive result. Although several studies with small number of patients have shown that Rituximab (anti-CD20 IgG) therapy can significantly improve the secretion function of the exocrine glands in pSS patients (4–6), a very recent large randomized controlled trial of Rituxmab did not confirm the effectiveness (7). In addition, since most B cell targeting therapies target CD20 (8), they are unable to deplete autoantibody-producing plasma cells, making it difficult to use this clinical evidence for reflecting the role of autoantibodies in pathogenesis of pSS.

Animal models provide a powerful tool for understanding the pathogenesis of disease. So far, several mouse models for pSS have been established and they provide some evidence for the role of autoantibodies in the disease pathogenesis (9). An essential role of B cells and autoantibodies in the impairment in secretion function of exocrine glands has been demonstrated NOD mouse model, where NOD.Igμ^null^ mice show no impairment in the secretion function (10). In addition, two mouse lines, mice overexpressing B cell activating factor and mice deficient in ACT1, a negative regulator of B cell survival, develop a pSS-like disease spontaneously (11, 12), also supporting an important role of B cell in the pathogenesis of experimental pSS. By contrast, in the *Id3−/−* mouse model (13) and the M3R immunization-induced mouse model (14), transfer of purified autoreactive T cells has been shown to be sufficient for inducing pSS-like symptoms, arguing that B cells and autoantibodies are dispensable in their pathogenesis. Since pSS is an autoimmune syndrome of which pathogenesis might differ from patient to patient, one animal model can only represent pathogenesis of a small part of patients. Therefore, to better explore the role of B cells, more animal models are required.

In 2013, Jonsson et al. reported that the presymptomatic presence of anti-SSA/Ro autoantibodies shows the highest odds ratio for the risk of development of pSS, followed by anti-SSB/La autoantibodies and ANA (15). This finding identifies anti-SSA/Ro autoantibodies as a good predictive marker of pSS but also suggests that immune response against SSA/Ro antigen might play a role in the development of pSS. Since no evidence has been shown so far that anti-SSA/Ro autoantibodies itself have pathogenic properties (16), an alternative possibility is that autoimmune response to T cell epitopes within SSA/Ro antigen play an important role in the pathogenesis of pSS. This notion is supported by the evidence that autoimmune response against the predominant T cell epitope within Ro60 antigen can induce production of autoantibodies against multiple antigens *via* intermolecular epitope spreading (17). In the latter study, Deshmukh et al. determined the T cell epitopes of both human and murine Ro60 protein by immunization of mice with the intact protein and a subsequent evaluation of the T cell response directed against small synthetic peptides derived from the Ro60 sequence. Using this strategy, the authors identified several regions within human Ro60 containing T cell epitopes, including the hRo60_316-335 peptide. Regarding murine Ro60, the most dominant T cell epitope was identified within the mRo60_311-330 peptide, which overlapped hRo60_316-355 (17). Furthermore, immunizing mice with mRo60_316-335 peptide resulted in the generation of a variety of autoantibodies against multiple antigens, confirming that this peptide contains a dominant T cell epitope of mRo60 (17).

In this study, we hypothesized that autoimmune response to the T cell epitope of SSA/Ro antigen contribute to the pathogenesis of pSS by producing pathogenic autoantibodies *via* intermolecular epitope spreading. To verify this hypothesis and to establish a novel mouse model for pSS, we immunized mice with a murine Ro60_316-335 peptide containing the predominant T cell epitope of Ro60 antigen (17). Furthermore, we investigated the role of B cells in this novel mouse model of pSS.

## Materials and Methods

### Mice

All mice used in this study were female. C3H/HeJ, DBA/1J, and C57BL/6J mice were purchased from Shanghai SLAC laboratory Animal Co. (Shanghai, China), while C3H/HeN mice were purchased from Beijing Vital River Laboratory Animal Technology Co., Ltd. (Beijing, China). All mice were housed in the animal facility with a 12-h light–dark cycle at the Xiamen University. Mice were held at specific pathogen-free conditions and fed standard mouse chow and acidified drinking water *ad libitum*. Protocols of all animal experiments were approved by the Institutional Animal Care and Use Committee of Xiamen University.

### Peptides and Immunization

Murine Ro60_316-335 (KARIHPFHVLIALETYRAGH) peptides used in this study were synthesized at Research Center Borstel, Germany. Mice of the age from 8 to 10 weeks were immunized at the hind footpad with 100 µg peptide emulsified (1:1) in nonionic block copolymer adjuvant Titermax (Alexis Biochemicals, Lorrach, Germany). Control mice were treated with PBS emulsified in Titermax. The mice were followed until 12 weeks after immunization.

### Measurement of Saliva and Tears

Mouse saliva and tears were measured at week 0, 6, and 12 after immunization as described previously (18), with slight modification. Briefly, mice were starved for 16– h before the measurement, deeply anesthetized, and stimulated by pilocarpine hydrochloride (0.5 mg/kg body weight) (Sigma-Aldrich). Saliva was collected with a sponge immediately after the injection of the pilocarpine for a duration of 20 min. Tears was collected using Phenol Red Thread (Jingming Ltd., Tianjin, China) at the 10 and 20 min time points after the injection of the pilocarpine. Since the body weight of mice increased during the time of the experiments (e.g., from 18.6 to 21.7 g mean body weight of C3H/HeJ mice during 12 weeks), both saliva and tears secretion volumes were normalized to their individual body weight.

### Histopathological Assessment and Immunohistochemistry Staining

Histology of exocrine glands or kidney was evaluated by Hematoxylin and Eosin (H&E) staining with 6-μm-thick sections prepared from formalin-fixed tissues. Immunochemistry in exocrine gland specimens was performed using antibodies against murine CD3 (ab5690, Abcam) and B220 (RA3-6B, eBioscience) with 6-μm-thick paraffin sections.

### Enzyme-Linked Immunosorbent Assay

An enzyme-linked immunosorbent assay (ELISA) was used to detect antibodies against mRo60_316-335 peptides. The SSA peptides (10 mg/ml in 0.5 M Na_2_CO_3_ buffer; pH 9.6) were absorbed onto Costar EIA/RIA Plates (Corning Incorporated, Corning, NY, USA), washed and blocked with 3% BSA in PBS with 0.05% Tween-20 (PBS-T), incubated with the respective mouse sera (1:200), and further washed with PBS-T. Bound antibodies were detected using peroxidase conjugated goat anti-mouse IgG antibodies (Sigma, USA) and tetramethylbenzidine (Solarbio, Beijing, China). Concentrations of IFN-γ, IL-17A, IL-4, and IL-10 in mouse sera were determined by commercially available ELISA kits (Peprotech, USA) according to the manufacturer’s protocols. The detection range of the assay for IFN-γ, IL-17A, IL-4, and IL-10 was 7.5–1,000 pg/ml, 7.5–1,000 pg/ml, 40–5,000 pg/ml, and 15–2,000 pg/ml, respectively. All sera were diluted 1:3 before quantification.

### Immunoblotting

Samples of total proteins extracted from lysates of salivary or lacrimal glands from healthy C57BL/6J mice were subjected a 10% SDS-PAGE gel and then separated proteins were transferred to PVDF membranes. Membranes were blocked using 5% de-fated milk in PBS-T for 2 h at room temperature. After washing, membranes were incubated with mouse sera (1:200 dilution in blocking buffer) from immunized or control mice overnight at 4°C and subsequently incubated with peroxidase conjugated goat anti-mouse IgG antibodies for 1 h at room temperature followed by visualization of bands using chemiluminescent substrate.

### *In Vivo* B Cell Depletion

To deplete B cells, we injected C3H/He mice i.v. with 10 mg/kg anti-CD20 antibody 1 day before the immunization. To maintain the depletion, the same dose of anti-CD20 antibody was injected at the third, sixth, and ninth week after the immunization. Both anti-CD20 monoclonal antibody (18B12) and isotype control IgG were kind gift from Biogen (Biogen Inc., San Diego). Efficiency of depletion of the B cells was evaluated by determining B cells in the peripheral blood using FITC-conjugated anti-mouse CD19 IgG (6D5, Biolegend, USA).

### Epitope Prediction and Sequence Similarity Search

The protein sequence of *Homo sapiens* Ro60 ribonucleoprote in isoform 3 (NP_001035829.2) was retrieved from the NIH database (http://www.ncbi.nlm.nih.gov/protein). By using the IEDB Analysis Resource Consensus tool (19), retrieved data were used to develop a prediction model that could identify the peptides binding to pSS-associated HLA-DR1*0803 allele. The percentile rank was applied for the output, and the binding affinity to the epitope was artificially set as 1/percentile rank. Amino acid sequence similarity search was performed by BLASTP software (https://blast.ncbi.nlm.nih.gov/).

### Statistical Analysis

All analyses were performed with GraphPad Prism statistical software (GraphPad Software Inc., version 5.01, La Jolla, CA, USA). Normality and equality of variances of the quantitative data was determined first. At *P* values > 0.05, data were considered as normally distributed with equal variances. Depending on the quality of the data, data with normal distribution were further analyzed by unpaired Student’s *t*-test with or without Welch’s correction, while data displaying a non-parametric distribution were analyzed by using Mann–Whitney *U* test. *P* values below 0.05 were considered as statistically significant.

## Results

### Induction of a pSS-Like Disease in C3H/HeJ Mice by Immunization with a Ro60-Derived T Cell Epitope

**Figure 1:**
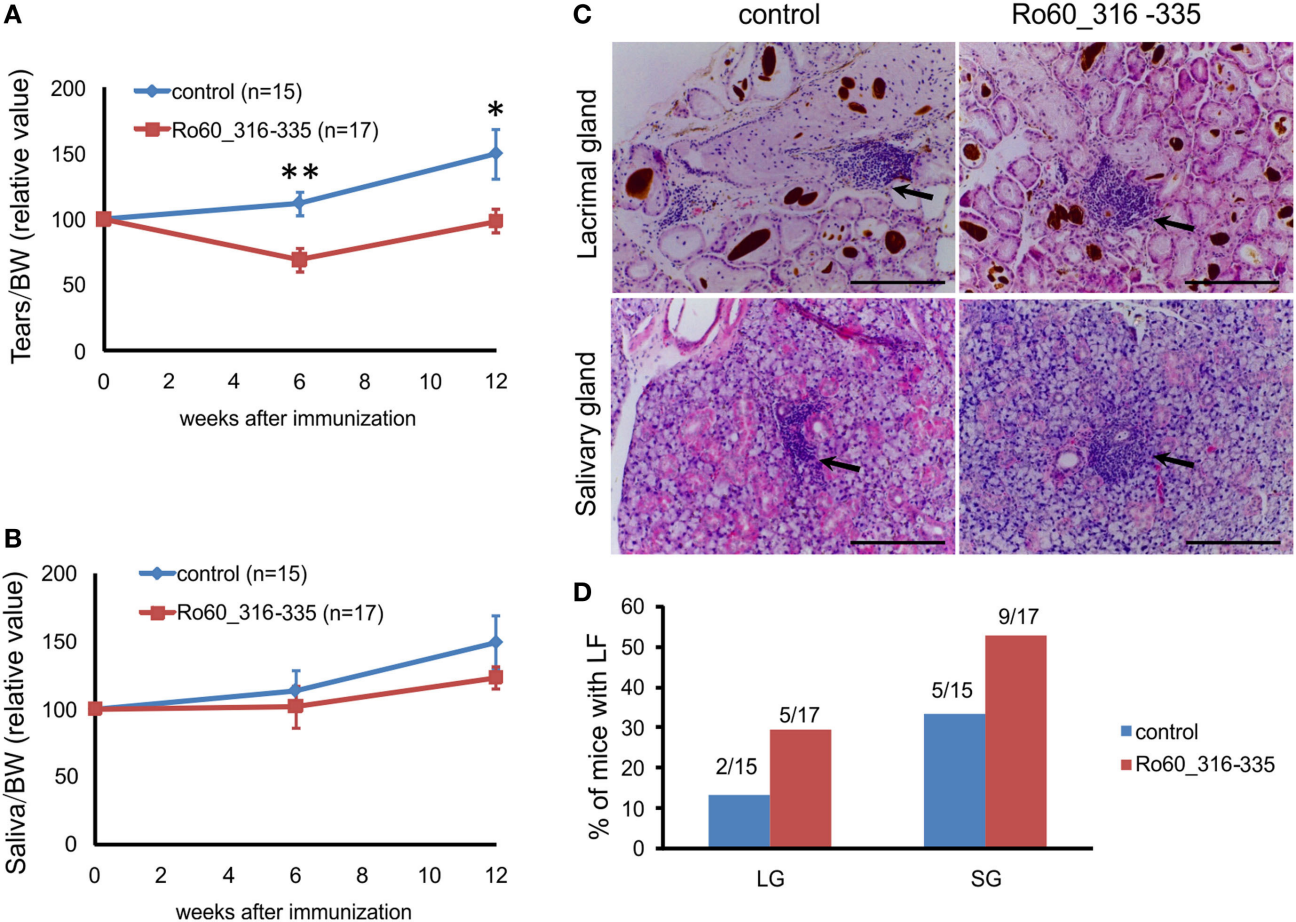
Immunization with the Ro60_316-335 peptide induces a primary Sjögren’s syndrome-like disease in C3H/He mice. C3H/HeJ mice were immunized with Ro60_315-336 (*n* = 17) or treated with PBS as control (*n* = 15) and secretion of tears **(A)** and saliva **(B)** was determined after pilocarpine stimulation. Values were normalized to the respective body weights and subsequently to the levels of secretion determined before immunization. Results two experiments were pooled and data are presented as mean ± SEM. Statistically significant differences between peptide-immunized mice and controls were calculated by using the Mann–Whitney *U*-test (*p < 0.05 and **p < 0.01). **(C)** Representative sections with lymphocytic foci (LF) derived from lacrimal (upper panel) and salivary glands (lower panel) of Ro60_315-336 immunized mice or controls after Hematoxylin and Eosin staining. Black arrows indicate LF. Bars, 100 µm. **(D)** Incidence of mice with LF in lacrimal and salivary glands. Numbers above bars indicate the ratio of number of mice with LF/total number of mice examined.

Previously, Deshmukh and colleagues reported that the predominant T cell epitope of mRo60 antigen is located within mRo60_316-335 peptide (17). We first investigated whether immunization with the mRo60_316-335 peptide could induce a pSS-like disease in mice. As described by others (13), tear secretion in control mice typically increased during the life span of the animals. As compared to the controls, C3H/HeJ mice immunized with mRo60_316-335 peptide showed a decreased production of tear at both 6 and 12 weeks after immunization (Figure 1A), suggesting an impaired function of the tear secretion. However, no significant difference was observed in the secretion of saliva between the two groups (Figure 1B).

Lymphocytic foci (LF), a hallmark of pSS, were observed in both lacrimal and salivary glands (Figure 1C). Unexpectedly, LF were observed in both groups of experimental mice, with a none-significant trend of higher incidence in Ro60_316-335 peptide-immunized mice (9 out of 17 in salivary gland and 5 out 17 in lacrimal gland) than in controls (5 out of 15 in salivary gland and 2 out 15 in lacrimal gland) (Figure 1D).

**Figure 2:**
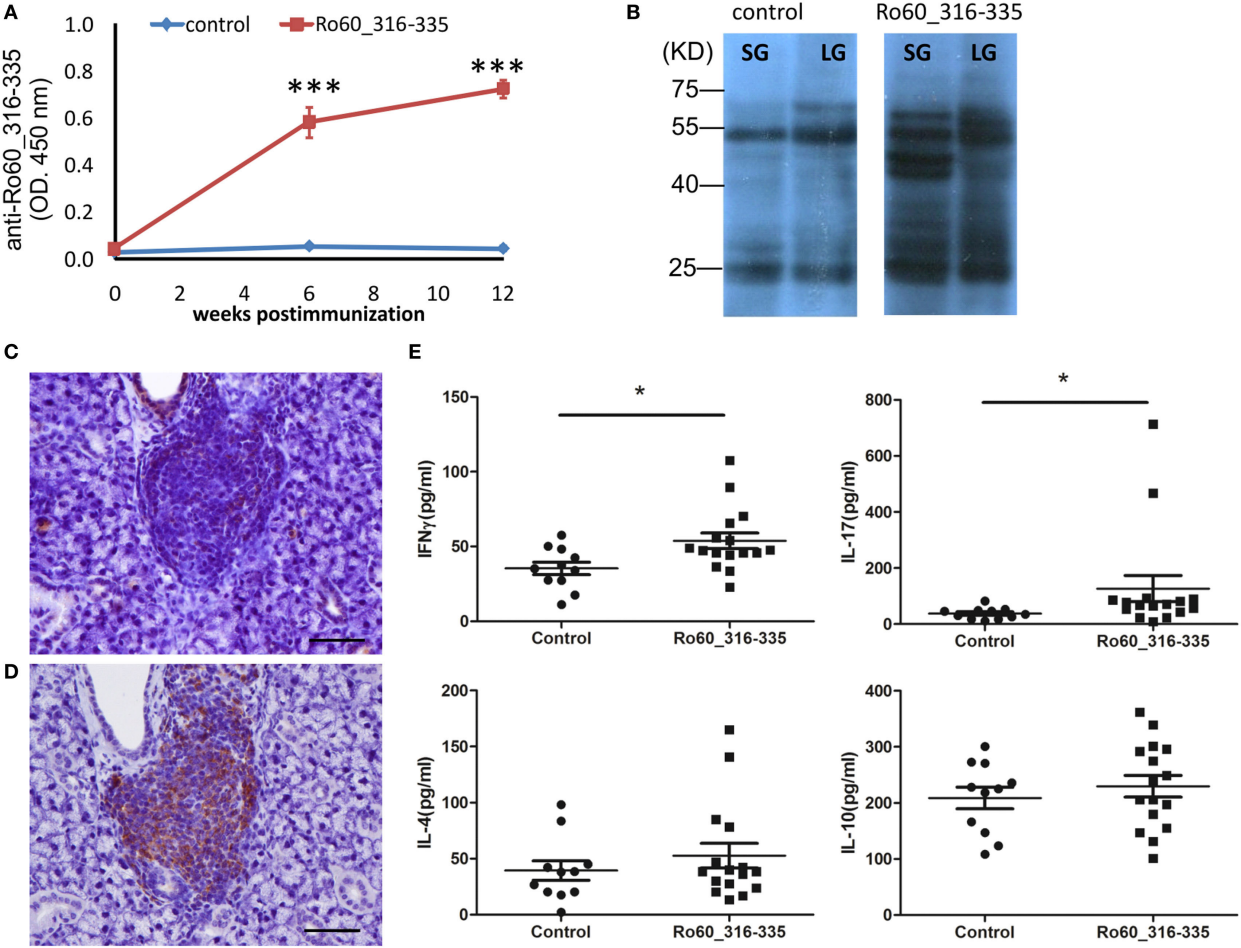
Immunological features of the primary Sjögren’s syndrome-like disease in C3H/He mice. **(A)** Autoantibodies against the Ro60_316-335 peptide in sera of Ro60_316-335 immunized (*n* = 9) C3H/HeJ mice or controls (*n* = 9). Results derived from one of two experiments performed are shown and data are presented as mean ± SEM. Statistically significant differences (*p < 0.05 and **p < 0.01) were calculated by using the Mann–Whitney *U*-test. **(B)** Immunoblotting of lacrimal glands (LG) or salivary glands (SG) tissue lysates from healthy mice with sera from control or Ro60_316-335 immunized mice. Representative results are shown. **(C,D)** immunochemistry staining of murine CD3 T cells **(C)** and CD19 B cell **(D)** in the lymphocytic foci (bar length = 50 µm). **(E)** Concentrations of IFN-γ, IL-17A, IL-4, and IL-10 in the sera of Ro60_316-335 immunized C3H/HeJ mice (*n* = 16) and controls (*n* = 11). Statistically significant differences (*p < 0.05) were calculated by the Mann–Whitney *U*-test.

In the next step, we investigated the production of autoantibodies. As expected, C3H/HeJ mice immunized with the mRo60_316-335 peptide generated autoantibodies against the antigen while corresponding control mice did not (Figure 2A). To determine the interaction of serum-autoantibodies with glandular proteins, binding of antibodies to protein extracts derived from lacrimal or salivary glands of healthy mice were analyzed by western blot and bands were visualized by using a second antibody to murine IgG. While blots developed with sera from control mice showed only two major binds which corresponds to the heavy and light chains of murine IgG, sera from mRo60_316-335 peptide-immunized C3H/HeJ mice displayed multiple bands, suggesting that sera from mRo60_316-335 peptide-immunized C3H/HeJ mice contained multiple autoantibodies against exocrine glands-derived proteins (Figure 2B). Furthermore, immunochemistry staining of the salivary gland demonstrated that the LF were composed of both T cell and B cells, with a predominance of B cells (Figures 2C,D). Finally, we determined the serum levels of T cell associated cytokines, including IFN-γ, IL-17A, IL-4, and IL-10. Sera of C3H/HeJ mice immunized with mRo60_316-335 showed significantly higher levels of IFN-γ and IL-17A as compared to control mice, while no significant difference in the levels of IL-4 or IL-10 were observed between both groups (Figure 2E).

Since SSA autoantibodies are also found in systemic lupus erythematosus (SLE), we examined whether immunized mice express SLE-like symptoms. No obvious difference was observed in the H&E staining of kidney sections between the mRo60_316-335 peptide immunized mice and controls. In consistence with this, no significant difference was found in the concentration of proteins in the urine of the two groups of mice (Figure S1 in Supplementary Material). Therefore, immunization with Ro60_316-335 peptide induced a pSS-like disease in both, C3H/HeJ and C3H/HeN mice.

### Development of the pSS-Like Disease among Mouse Strains

Since genetic background plays an important role in the development of experimental autoimmune disorders, we then investigated the development of the Ro60_316-335 peptide-induced pSS-like disease in other mouse strains. We first investigated C3H/HeN mice, a strain closely related to C3H/HeJ. Six weeks after immunization, C3H/HeN mice treated with the mRo60_316-335 peptide showed a significant decrease in the production of tears as compared to control mice (Figure S2A in Supplementary Material). No significant difference was observed in the secretion of saliva between the two groups (Figure S2B in Supplementary Material). Similar to C3H/HeJ mice, C3H/HeN mice immunized with mRo60_316-335 peptide generated autoantibodies against the peptide and protein extracts from lacrimal and salivary glands (Figure S2C,D in Supplementary Material). With regard to inflammatory cell infiltration, in C3H/HeN mice, LF were almost exclusively observed in Ro60_316-335 peptide-immunized mice but not in control mice, although the frequency of mice with LF was rather low (Figures S2E,F in Supplementary Material).

We next investigated other two mouse strains carrying different MHC allele, DBA1/J and C57BL/6J mice. DBA/1J mice treated with mRo60_316-335 peptide did not show any impairment in secretion function of salivary or lacrimal glands as compared to control mice. Furthermore, neither LF nor infiltrated cells was observed in the immunized mice. Although autoantibodies against Ro60_316-335 peptide can be detected in sera of DBA/1J mice treated with the peptide, no autoantibodies against proteins from lachrymal and salivary glands were detectable. In addition, serum levels of IL-17A but not IFN-λ, IL-4, or IL-10 was increased in peptide immunized mice as compared to control mice (Figure S3 in Supplementary Material). Similar to DBA/1J mice, C57BL/6J mice were also resistant to the Ro60_316-335 peptide-induced pSS-like disease, without impairment in secretion of tears, infiltration of lymphocytes in exocrine glands or production of autoantibodies binding to proteins from exocrine gland (Figure S4 in Supplementary Material).

Table 1 summarizes the disease symptoms and immunological features in four tested mouse strains. Besides impairment in tears secretion, there are two major differences between susceptible C3H/He strains, and resistant strains, DBA/1J and C57BL/6J. One is that susceptible mice produced autoantibodies against proteins of the exocrine glands but resistant mice did not. The other is that lymphocytic infiltration into exocrine glands was observed in the susceptible mice but not resistant mice.

**Table 1:** Summary of the development of the Ro60_316-335 induced primary Sjögren’s syndrome -like disease among mouse strains.

	C3H/HeJ	C3H/HeN	DBA/1J	C57BL/6J
MHC II hyplotype	H2-d	H2-d	H2-q	H2-b
Tears secretion	Decreased	Decreased	Not affected	Not affected
Saliva secretion	Not affected	Not affected	Not affected	Not affected
Lymphocytic foci	Yes	Yes	No	No
Inflammatory cell infiltration	Yes	Yes	No	No
anti-Ro60_316-335 IgG	Yes	Yes	Yes	Yes
anti-exocrine gland lysate IgG	Yes	Yes	No	No
IFN-γ	Increased	–	Not affected	Increased
IL-17A	Increased	–	Increased	Not affected

*–, not evaluated*.

### B Cell-Depletion Prevents the Development of pSS-Like Disease in C3H/He Mice

**Figure 3:**
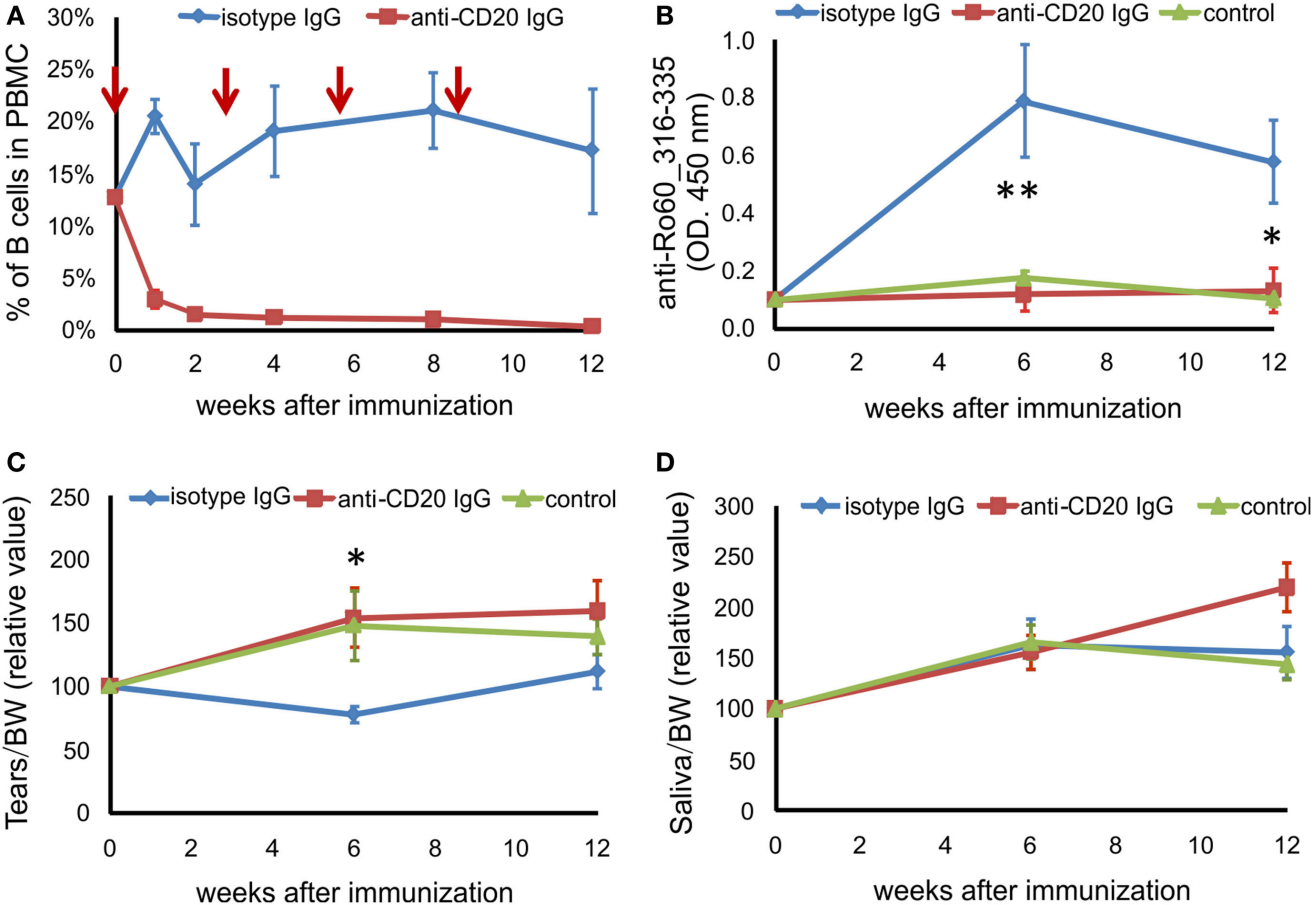
B cell depletion prevents mice from Ro60_315-336-induced primary Sjögren’s syndrome-like disease. Before and after immunization with Ro60_316-335 peptide, C3H/HeN mice were injected i.p. with anti-CD20 IgG (red line) or isotype IgG (blue line). The control group (green line) represents mice immunized with PBS and adjuvants alone. **(A)** Efficiency of depletion of murine B cells in C3H/HeN mice. The time points of B cell-depleting anti-CD20 antibody injection are indicated by red arrows, the percentage of B cells in the peripheral blood mononuclear cells were determined at 0, 1, 2, 4, 8, and 12 weeks after immunization using CD19 as marker for B cells. **(B)** Autoantibodies against the mRo60_316-335 peptides in sera of isotype IgG treated (*n* = 7) or anti-CD20 IgG treated (*n* = 8) mRo60_316-335-immunized C3H/HeN mice, or PBS-treated control mice (*n* = 6). Tears **(C)** and saliva **(D)** production in isotype IgG treated (*n* = 7) or anti-CD20 IgG treated (*n* = 8) mRo60_316-335-immunized C3H/HeN mice, or control mice (*n* = 6). Data are presented as mean ± SEM. Statistically significant differences between isotype IgG (*n* = 7) or anti-CD20 IgG treated (*n* = 8) mice were calculated by the unpaired Student’s *t*-test with Welch’s correction (*p < 0.05, **p < 0.01, unpaired Student’s *t*-test).

We next investigated the role of B cells in this novel mouse model of pSS. C3H/HeN mice were injected i.p. with anti-CD20 antibody before immunization as well as at third, sixth, and ninth weeks after immunization. We evaluated the efficiency of B cell depletion by detecting B cells in peripheral blood. As shown in Figure 3A, the percentage of CD19 + B cells in peripheral blood mononuclear cell decreased from 12.67% before depletion to 2.94% 1 week after the first anti-CD20 injection, further decreased to less than 1.5% 2 weeks after the anti-CD20 injection, and maintained at a level of lower than 1.5% during the whole experiment period, while the B cell levels in mice treated with isotype IgG did not change significantly (Figure 3A). Furthermore, after immunization with Ro60_316-335 peptide, mice treated with anti-CD20 antibodies did not produce autoantibodies against the peptide, while animals which received irrelevant antibodies of the same isotype did (Figure 3B), confirming that B cells were depleted efficiently. Clinically, mice treated with isotype IgG showed a decreased production of tears at 6 weeks after the immunization with Ro60_316-335 peptide, while this impaired secretion function was not observed in mice treated with anti-CD20 IgG (Figures 3C,D), suggesting that B cell-depletion prevented the impairment in the tears secretion.

### Prediction of Epitope within hRo60 for pSS-Associated HLA-DRB1 Alleles

**Figure 4:**
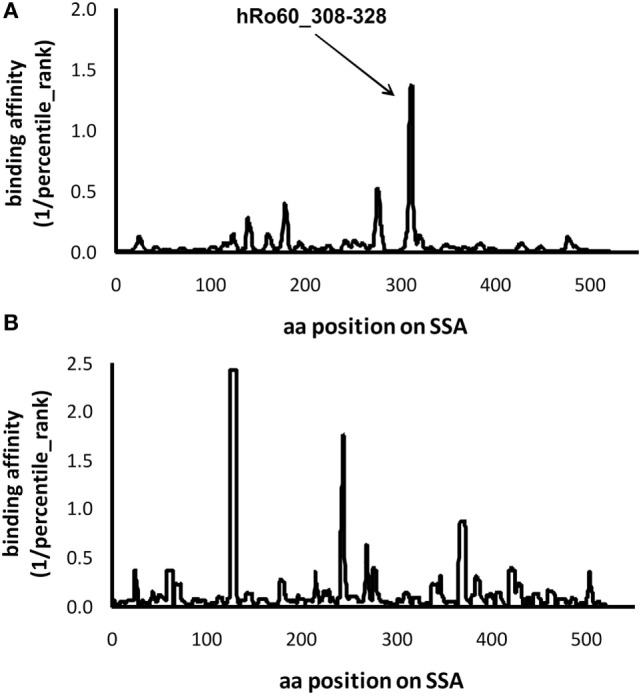
Prediction of the epitopes for the primary Sjögren’s syndrome (pSS)-risk associated HLA-DRB1 alleles within the hRo60 protein. The protein sequence of *Homo sapiens* Ro60 ribonucleoprote in isoform 3 (NP_001035829.2) was retrieved from the NIH database (http://www.ncbi.nlm.nih.gov/protein). By using the IEDB Analysis Resource Consensus tool, retrieved data were used to develop a prediction model that could identify the peptides binding to pSS-associated HLA-DRB1*0803 **(A)** and HLA-DRB1*0301 **(B)** alleles. The percentile rank was applied for the output, and the binding affinity to the epitope was artificially set as 1/percentile rank.

Our data suggest a pathogenic role of the predominant T cell epitope of Ro60 in the development of pSS-like but not SLE-like disease in mice. Previously, we performed a comprehensive meta-analysis and demonstrated that HLA-DRB1*0301 and HLA-DRB1*0803 are two major alleles associated with an increased risk of pSS (20). We then investigated whether these two pSS-associated HLA alleles could bind the T epitopes within human Ro60 antigen using IEDB Analysis Resource Consensus tool. Within human Ro60, there is a predominant T cell epitope located within the amino acid sequence of 308aa–328aa (Figure 4), a region which has been shown to contain a T cell epitope of hRo60 (17). Another pSS associated allele, HLA-DRB1*0301, was predicted to bind three strong T cell epitopes within the hR60 antigen (Figure 4).

## Discussion

In this study, we induced a pSS-like disease by immunizing mice with a partial structure derived from the mRo60 protein, a peptide containing the predominant T cell epitope. Previously, Scofield et al. established a mouse model of pSS by repetitive immunization with Ro60 peptide containing predominant B cell epitopes (21). In the current study, susceptible mice developed pSS-like disease with only single immunization with peptide containing predominant T cell epitope, indicating that immunization with a T cell epitope might be more effective than a B cell epitope in induction of the disease. T cell epitopes within disease-associated autoantigens have been already identified in many systemic autoimmune disorders, e.g., SSc-associated topoisomerase I (22, 23) or SLE-associated histones (24). Therefore, the experimental approach in our current study might provide a good strategy for establishing further mouse models for different systemic autoimmune diseases.

**Figure 5:**
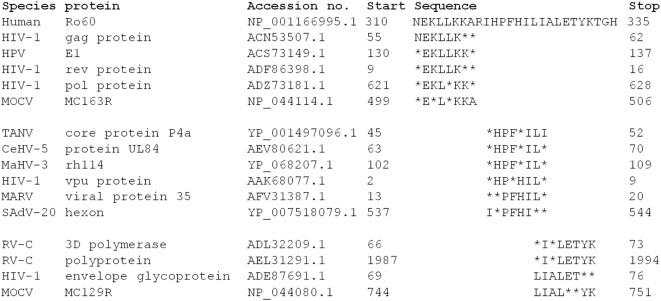
Analysis of the sequence homology between hRo60_310-335 and human virus proteins. Sequence homologies were analyzed by BLASTP software (https://blast.ncbi.nlm.nih.gov/).

The Ro60_316-335 peptide induced novel mouse model of pSS demonstrates that T cell epitope in this region is pathogenic in mice. Moreover, a T cell epitope in this section is predicted to bind to the pSS-risk associated HLA-DRB1*0803 allele, a HLA allele associated with pSS but not SLE (25), indicating that such epitope could indeed play a role in the development of pSS. Viral infections are currently seen as one potential risk factor in the development of autoimmune disease, including pSS (26). A reason for this may be sequence similarities between viral and host proteins leading to an unwanted immune response by molecular mimicry. A search of the sequence similarity between virus proteins and the hRo60 revealed that proteins from multiple virus proteins share five to six continuous amino acid residues with hRo60_310-335 peptide (Figure 5). Among those virus, human immunodeficiency virus type 1 (HIV-1) may be of specific interest since several lines of epidemiological and serological evidence suggest that HIV-1 represents a triggering factors for the development of SS (27, 28). Thus, a possible link between the HIV-1 infection and SS disease could be based on molecular mimicry.

As expected, different mouse strains show diverse susceptibility to these novel mouse models of pSS, which might reflect the situation in human where genetic factor contribute to the development of disease (29, 30). Therefore, examining differences among susceptible and resistant mouse strains can provide hints for understanding the pathogenesis of this mouse model. Notably, susceptible strains immunized with the mRo60_316-335 peptide did not only develop autoantibodies directed against the peptide but also some autoantibodies with a specificity unrelated to the antigen, indicating intermolecular antigen spreading (17). Since these autoantibodies produced *via* intermolecular antigen spreading were not observed in disease-resistant strains, these observations support our hypothesis that immunization with a T cell epitope might lead to intermolecular antigen spreading and to a consequent production of potentially pathogenic autoantibodies against further autoantigens which may mediate disease symptoms. Moreover, the susceptible strains carry H2-k (C3H/HeJ and C3H/HeN) in the MHC locus, while the resistant strains carry H2-q (DBA/1J) or H2-b (C57BL/6J), supporting the important role of the HLA in the development of the disease (20, 31). In addition, only susceptible strains but not resistant strains are characterized with lymphocytic infiltration in the exocrine glands, indicating a contribution of infiltrated lymphocytes.

Despite the similarities in the pathogenesis of experimental pSS in C3H/HeJ and C3H/HeN, it should be noted that there are some fine differences in the disease phenotypes developed in both substrains. For example, C3H/HeJ mice showed a higher level of inflammatory infiltration in exocrine glands in both Ro60_316-335 peptide immunized and PBS treated groups than C3H/HeN mice. Since the only known relevant genetic difference between these closely related substrains can be referred to the expression of TLR4, which is absent in C3H/HeJ but present in C3H/HeN mice (32), a contribution of TLR4 to the pathogenesis of pSS can be suggested.

Notably, in this new model, the mice developed specifically pSS-like but not SLE-like symptoms. In humans, the anti-SSA/Ro60 antibodies have been detected in the presymptomatic phases of both, pSS and SLE (15, 33). Our model provides a first hint to explain the fine difference between pSS and SLE in the immune response to SSA/Ro60. Previously, the observed difference between both disorders was explained by disease-specific B cell epitopes (34). According to this hypothesis, autoantibodies against hRo60_169-190 should be specific for SLE and hRo60_211-232 for pSS (34). However, this idea was in contrast to further studies performed with larger numbers of samples (35). Results from our study provide a new explanation here: instead of disease-specific B cell epitopes, disease-specific T cell epitope on the Ro60 antigen may be responsible for the development of disease-specific pathologies.

Perhaps the most important finding of this study is that B cell-depletion prevents the impairment of secretion function of exocrine glands. However, this result may have two reasons. In autoimmune responses, B cells are involved in two essential processes, the presentation of antigen to T cells and producing autoantibodies. Since B cells were depleted before immunization and, thus, both processes were potentially affected, we are currently unable to delineate the individual contribution of each mechanism to the disease development. To address this issue, B cell depletion at different time points after the initiation of the immune responses will help to explore the underlying mechanism. The essential role of B cell in this novel mouse model of pSS provides some helpful evidence for clinical treatment of the human disease because it strongly argues for therapeutic approaches targeting B cells. Therefore, although the efficacy of current therapeutics targeting B cells have been proved not to be consistently effective (4, 5, 7), therapeutic approaches targeting plasma cells or autoantibodies might be of interest (36).

It should be mentioned that our model does not cover the entire pathology of pSS. First, disease symptoms are rather mild and impairment in secretion is observed only for tear but not for salivary glands. Second, although the formation of LF as a hallmark of pSS was observed in susceptible mice after immunization, no significant difference in incidence of LF between peptide-immunized mice and controls occurred. Finally, although the incidence of LF in salivary gland was higher than that in lacrimal glands, saliva production was found not to be impaired in this setting. This result argues against a direct association between LF development and secretion function which is not consistent with findings for the pSS in humans.

In conclusion, in the current study, we have established a novel mouse model of pSS based on a single immunization with a Ro60 peptide containing a predominant T cell epitope, demonstrating that T cell epitope within SSA/Ro60 antigen is potentially pathogenic. Furthermore, our results support that B cells play an essential role in the development of pSS.

## Ethics Statement

All protocols of mouse experiments were approved by the Institutional Animal Care and Use Committee of Xiamen University.

## Author Contributions

XYu was involved in conception, design and supervision of the study. JZ, QH, RH, FD, XYue, YC, RH, WZ, LW and JY were involved in the performing experiment, acquisition of data and analysis of data. JZ, JY, and RH were involved in the bioinformatics analysis. XYu, GR, ZL and FP were involved in drafting of the manuscript.

## Conflict of Interest Statement

The authors declare that the research was conducted in the absence of any commercial or financial relationships that could be construed as a potential conflict of interest.
