# Supplementary material for: B Cells Are Indispensable for a Novel Mouse Model of Primary Sjögren’s Syndrome
Source: Front Immunol. 2017 Oct 24;8:1384. doi: 10.3389/fimmu.2017.01384 (PMC5661054; doi:10.3389/fimmu.2017.01384)
Supplement: Supplementary file 1 [file image_1.pdf]

Supplementary information

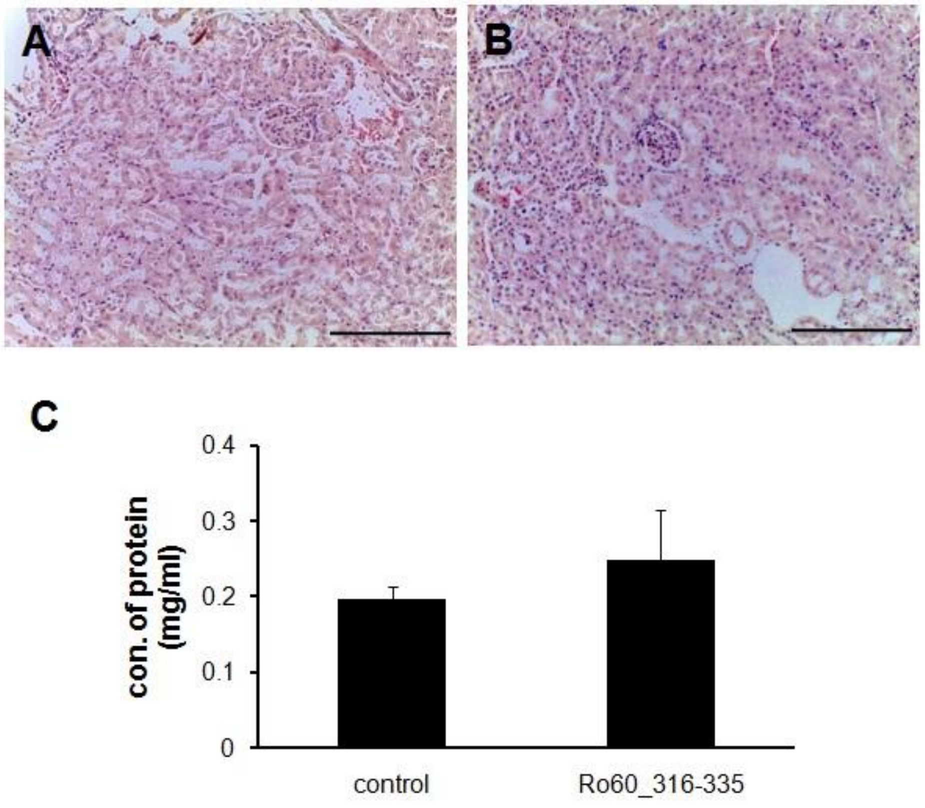

Supplementary figure 1: No disease symptoms in kidneys of mRo60_316-335 immunized C3H/He mice. Representative slides of H&E staining for kidney from control (A) and mRo60_316-335 immunized mice (B). Bars, 100 μm. (C). Protein in the urine of control and mRo60_316-335 immunized mice are not significantly different (Mann-Whitney U test). The concentration of protein in mouse urine was measured using Urine proteins test kit (Nanjing Jiancheng Bioengineering Institue, Nanjing, China) according to the provided protocol.

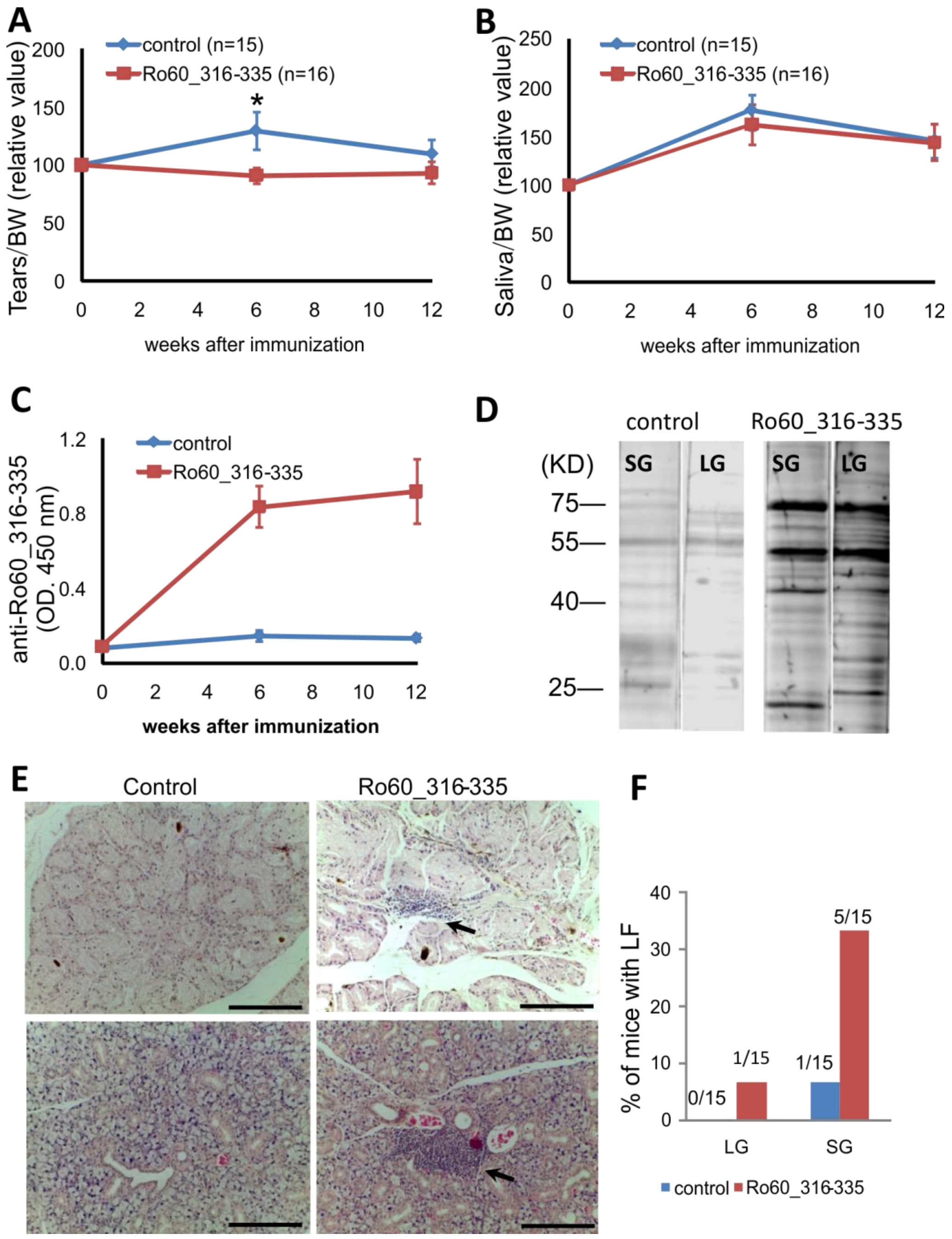

Supplementary figure 2. C3H/HeN mice are susceptible to Ro60_316-335 induced pSS-like disease. Secretion of tears (A) and saliva (B) in Ro60_316-335 peptide immunized mice (n=16) and PBS treated controls (n=15). Results two experiments were pooled and data are presented as mean ± SEM. Statistically significant differences were calculated by using the unpaired Student's *t*-test with Welch`s correction (* $p<0.05$). (C). Autoantibodies against the Ro60_316-335 peptide in sera of Ro60_316-335 immunized (n=8) mice or controls (n=9). Results derived from one of two experiments performed are presented as mean ± SEM. Statistical analyses were performed by the unpaired Student's *t*-test (*** $p<0.001$). (D). Immunoblotting of lacrimal glands (LG) or salivary glands (SG) tissue lysates from healthy

mice with sera from control or Ro60_316-335 immunized mice. (E). Representative sections derived from lacrimal (upper panel) and salivary glands (lower panel) of Ro60_315-336 immunized mice or controls after H&E staining. The black arrows indicate lymphocytic loci. Bars, 100 µm. (F). Incidence of mice with lymphoctic foci in lacrimal and salivary glands. Numbers above bars indicate the ratio of number of mice with LF/total number of mice examined.

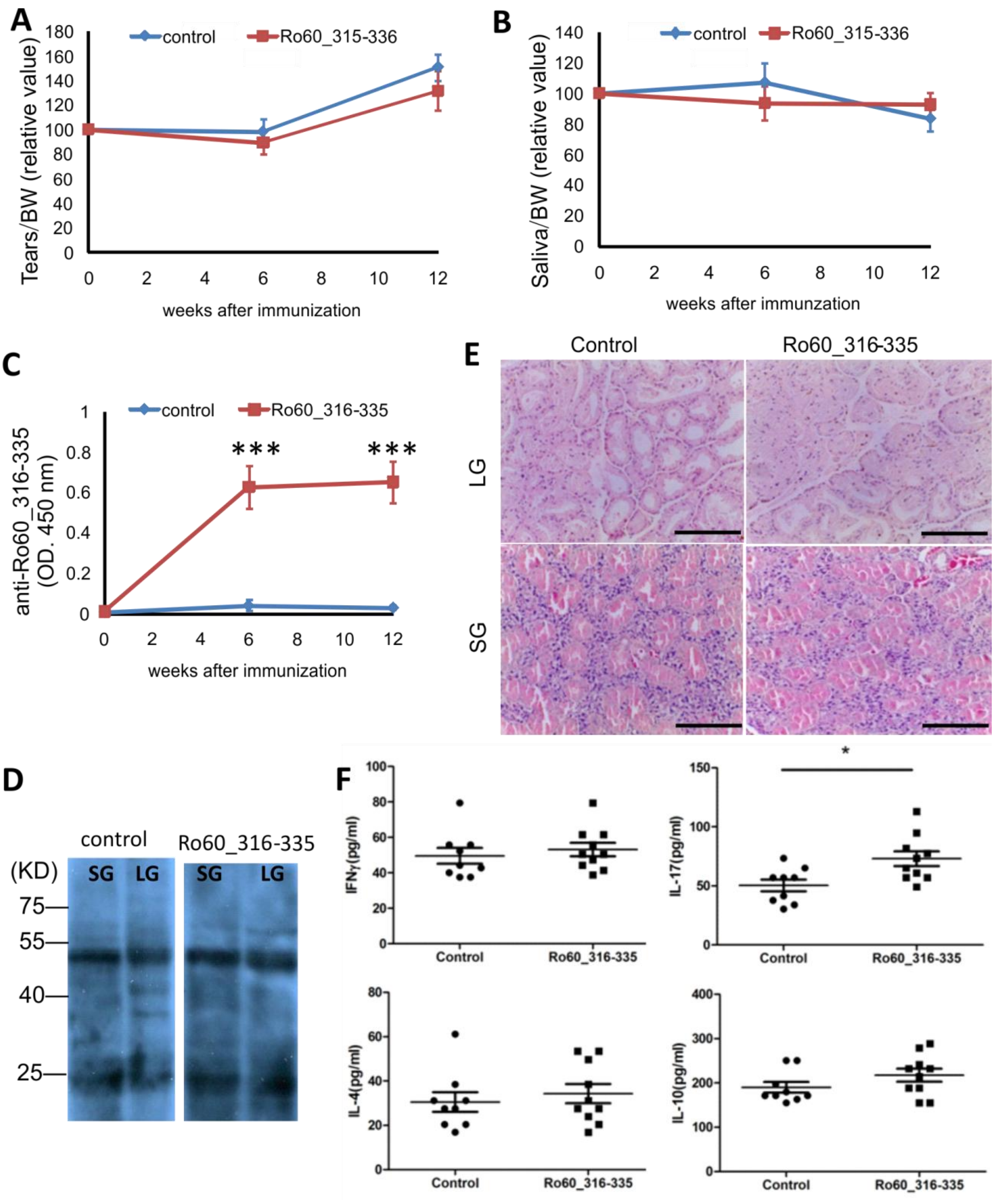

Supplementary figure 3. DBA/1J mice are resistant to Ro60_316-335 induced pSS-like disease. Secretion of tears (A) and saliva (B) in Ro60_316-335 peptide immunized mice (n=10) and PBS-treated controls (n=10). (C). Autoantibodies against the Ro60_316-335 peptide in sera of Ro60_316-335 immunized (n=10) mice or controls (n=10). Data are presented as mean ± SEM. Statistical analyses were performed by using the unpaired Student's *t*-test with Welch's correction (*** $P<0.001$). (D). Immunoblotting of lacrimal glands (LG) or salivary glands (SG) tissue lysates from healthy mice with sera from control or Ro60_316-335 immunized mice. (E). Representative sections derived from lacrimal (upper panel) and salivary glands (lower panel) of Ro60_315-336 immunized mice or controls after H&E staining (bar lenghts = 100 μm). (F). Levels of IFN-γ, IL-17A, IL-4 and IL-10 in the sera of Ro60_316-335 immunized mice (n=9) and controls (n=10). Data are presented as mean ± SEM. Statistical analyses were performed by the unpaired Student's *t*-test (*$p < 0.05$).

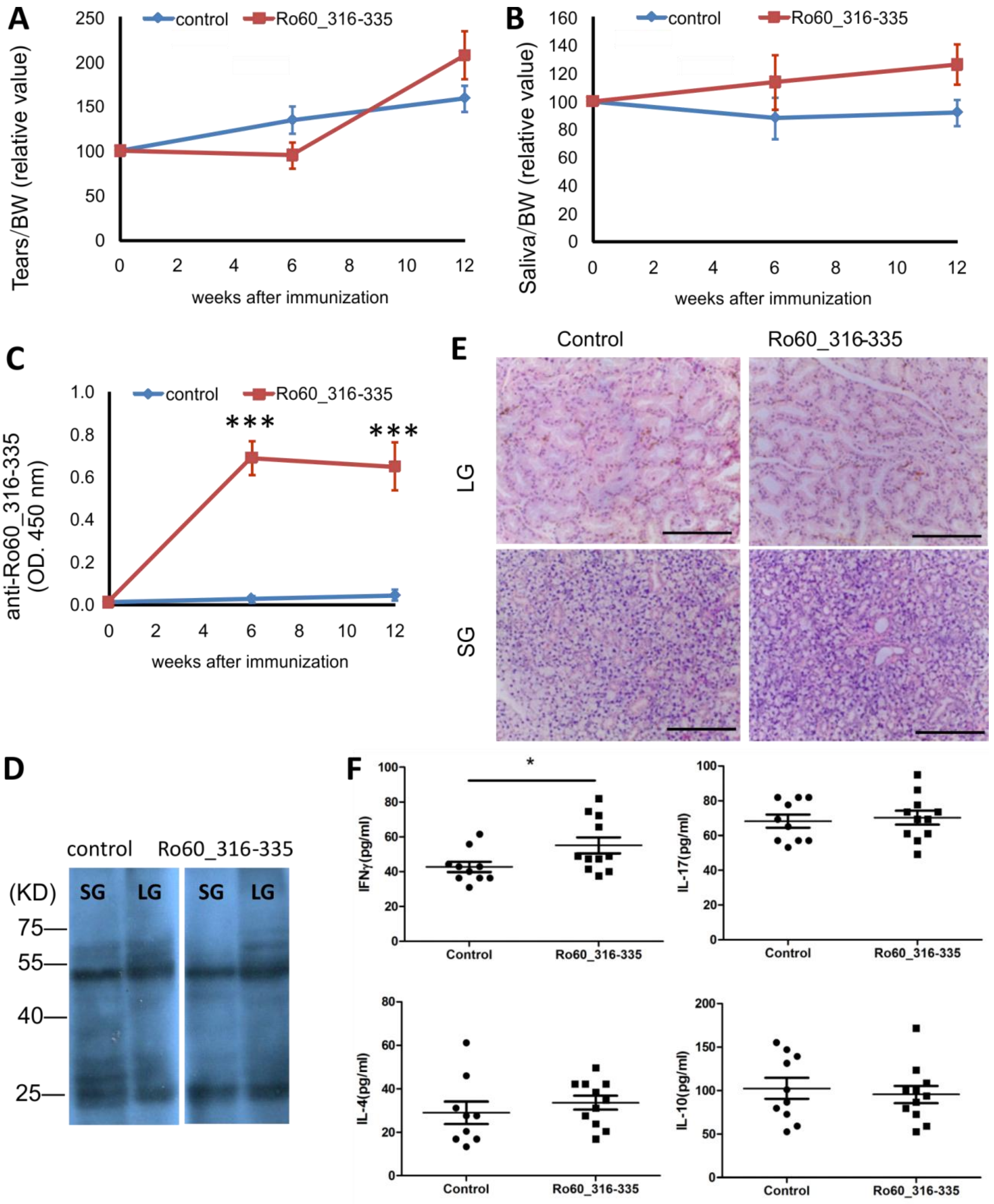

Supplementary figure 4. C57BL/6J mice are resistent to Ro60_316-335 induced pSS-like disease. Secretion of tears (A) and saliva (B) in mice immunized with mRo60_315-336 (n=14) or treated with PBS (n=12). Values were normalized to the respective body weights and subsequently to the levels of secretes determined before immunization. Data are presented as mean ± SEM. C. Autoantibodies against the mRo60_316-335 peptides in sera of mRo60_316-335 immunized (n=11) mice or controls (n=11). Data are presented as mean ± SEM. Statistical analyses were performed by using the unpaired Student's *t*-test with Welch's correction (*** $p<0.001$). (D). Immunoblotting of lacrimal glands (LG) or salivary glands (SG) tissue lysates from healthy mice with sera from control or mRo60_316-335 immunized

mice. Representative results are shown. (E). Representative sections derived from lacrimal (upper panel) and salivary glands (lower panel) of Ro60_315-336 immunized mice or controls after H&E staining (bar lenghts = 100 μm). (F). Levels of IFN-γ, IL-17A, IL-4 and IL-10 in the sera of Ro60_316-335 immunized mice (n=9) and controls (n=12). Statistical analyses were performed by using the unpaired Student's *t*-test with Welch's correction (*$p<0.05$).
